# Draft Genome Sequence of *Enterococcus faecalis* DD14, a Bacteriocinogenic Lactic Acid Bacterium with Anti-*Clostridium* Activity

**DOI:** 10.1128/genomeA.00695-17

**Published:** 2017-07-27

**Authors:** Yanath Belguesmia, Valérie Leclère, Matthieu Duban, Eric Auclair, Djamel Drider

**Affiliations:** a Université de Lille, INRA, Université d’Artois, Université du Littoral Côte d’Opale, EA 7394 Institut Charles Viollette, Lille, France; b Phileo Lesaffre Animal Care, Société Industrielle Lesaffre, Marcq-en-Barœul, France

## Abstract

We report the draft genome sequence of *Enterococcus faecalis* DD14, a strain isolated from meconium of a healthy newborn at Roubaix Hospital (France). The strain displayed antagonism against a set of Gram-positive bacteria through concomitant production of lactic acid and bacteriocin. The genome has a size of 2,893,365 bp and a 37.3% G+C ratio and is predicted to contain at least 2,755 coding sequences and 62 RNAs.

## GENOME ANNOUNCEMENT

Bacteria of the genus *Enterococcus* are fascinating to the scientific community and therefore are receiving a great deal of attention. These microbes are known to be adapted members of the gastrointestinal tracts (GITs) of humans and other mammals, as well as those of reptiles, birds, and insects (1). In addition, these bacteria are found in traditional fermented food and dairy products, water, soil, and plants (1). In contrast to lactobacilli, which are also abundant in the human GIT and associated with health benefits, enterococci are regarded as nosocomial pathogens and leading causes of a myriad of infections.

The word “enterococcus” dates back from the end of the 19th century with the description of a saprophytic and potentially infectious “coccus” of intestinal origin (2), Recently, Lebreton et al. (3) placed the origins of bacteria of the genus *Enterococcus* around the time of animal terrestrialization. *Enterococcus faecalis* is the most abundant species in the GIT, followed by *Enterococcus faecium* (4), but other species, including *Enterococcus avium* and *Enterococcus hirae*, are frequently found in human stool samples (4). Recently, *E. faecalis* DD14 was isolated from meconium (5) and characterized for its *Clostridium perfringens* leaderless two-peptide bacteriocin designated enterocin DD14 (6). Enterocins are safe bacteriocins (7) with possible applications as food additives and antibiotic-complementary and leishmanicidal agents (8–11).

The genome of *E. faecalis* DD14 was sequenced. The sequence data were obtained using Illumina MiSeq and HiSeq 2500 technology platforms, with 2- × 250-bp paired-end reads (MicrobesNG, Birmingham, United Kingdom). The closest available reference genome was identified using Kraken, and the reads were mapped to this genome using BWA mem (http://bio-bwa.sourceforge.net) to assess the quality of the data. *De novo* assembly of the reads was performed using SPAdes (http://bioinf.spbau.ru/spades). The functional annotation of predicted genes was achieved using the RAST server (http://rast.nmpdr.org) (12) to predict open reading frames (ORFs), followed by annotation using the SEED database (13). The draft genome of *E. faecalis* DD14, of 2,893,365 bp, includes 25 contigs with a 37.3% G+C ratio, 2,755 coding sequences, and 62 RNAs.

The *in silico* analysis of the genome of *E. faecalis* DD14 with the Bagel 3 tool (http://bagel2.molgenrug.nl) portrayed the presence of genes coding for peptide DD14A and peptide DD14B, respectively, which are highly similar to enterocin MR10A and MR10B (14). Another structural gene coding for enterolysin A was found in a cluster containing a putative associated immunity protein (15, 16). The DNA coding for polyribonucleotide nucleotidyltransferase was identified with the MultAlin tool (http://multalin.toulouse.inra.fr/multalin/) using the sequenced genome of *E. faecalis* V583 (GenBank gene identification no. 1201910). The Uniprot database (http://www.uniprot.org/uniprot/) entry is UniProtKB–Q82ZJ2 (PNP_ENTFA).

RAST analysis revealed vancomycin B-type resistance protein (VanW) (17), putative coding genes for resistance to β-lactamases and fluoroquinolones, genes coding for virulence factors such as aggregation substance (Asal), and siderophore component-coding DNA. Furthermore, the presence of a putative clustered regularly interspaced short palindromic repeat (CRISPR), which stands as a barrier to foreign DNA uptake, was identiﬁed in the first contig of *E. faecalis* DD14 by use of the CRISPR ﬁnder tool (18).

### Accession number(s).

The whole-genome sequence has been deposited GenBank under the accession no. CP021161. The version described in this paper is the first version, CP021161.1.
